# Penetrating Orbital Injuries: A Review

**DOI:** 10.7759/cureus.1725

**Published:** 2017-09-29

**Authors:** Faizullah Mashriqi, Joe Iwanaga, Marios Loukas, Anthony V D'Antoni, R. Shane Tubbs

**Affiliations:** 1 Department of Molecular, Cellular and Biomedical Sciences, CUNY School of Medicine; 2 Seattle Science Foundation; 3 Department of Anatomical Sciences, St. George's University School of Medicine, Grenada, West Indies; 4 Neurosurgery, Seattle Science Foundation

**Keywords:** orbit, pen, pencil, knife, penetrating injury, rim

## Abstract

Penetrating injuries to the orbit represent a small but very complicated portion of head injuries. Because of the close proximity to many vital structures, any penetrating orbital injury requires a multidisciplinary follow-up. Cases of penetrating injuries have flooded the literature, but no one has presented a systematic approach to the complications associated with these types of injuries. Herein, we present the complications associated with each orbital entry mode: superior, inferior, medial, lateral rims of the orbit, and extraorbital entry.

## Introduction and background

**Figure 1:**
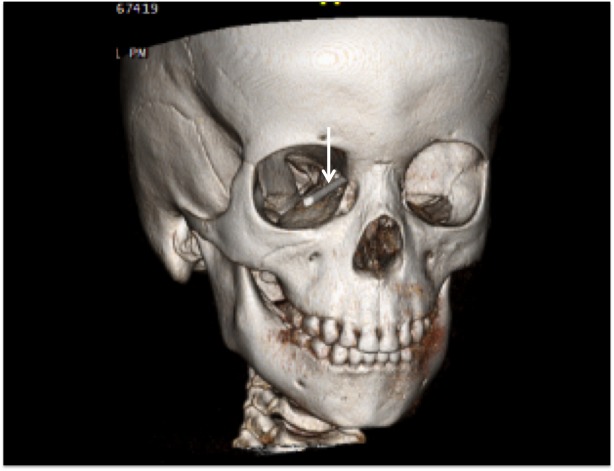
3D CT reconstruction of an adolescent who had fallen on a wooden stake (arrow). The stake entered the right orbit and traversed the ethmoid sinuses to become intracranial. 3D CT: three-dimensional computed tomography

**Figure 2:**
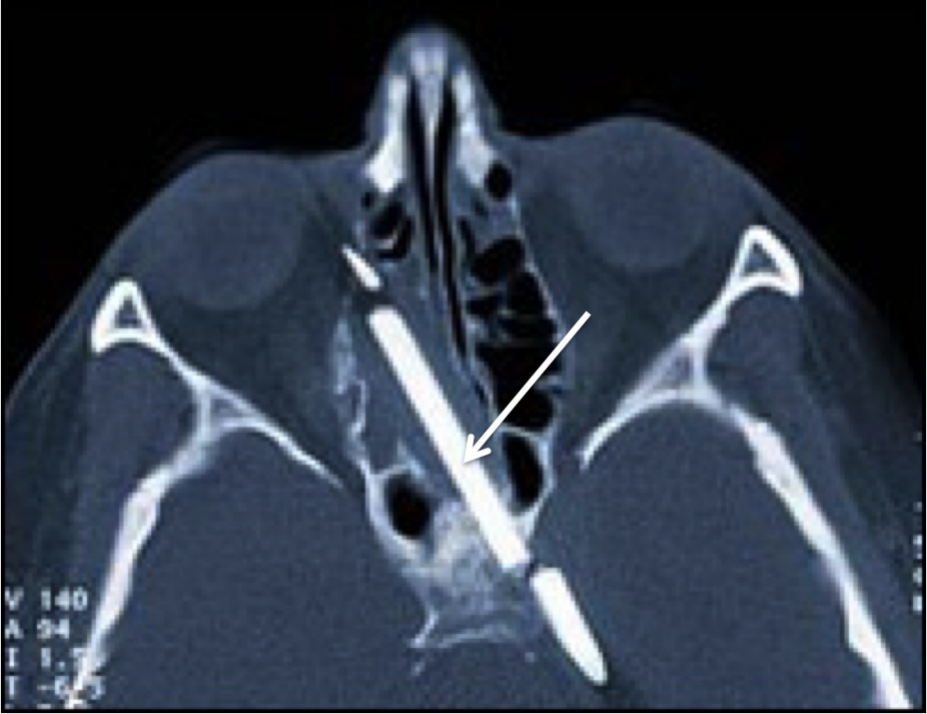
Axial head CT of a female child who fell onto a metal rod that entered the right medial orbit, traversed the ethmoid sinuses, and entered the left middle cranial fossa just lateral to the sella turcica. CT: computed tomography

**Figure 3:**
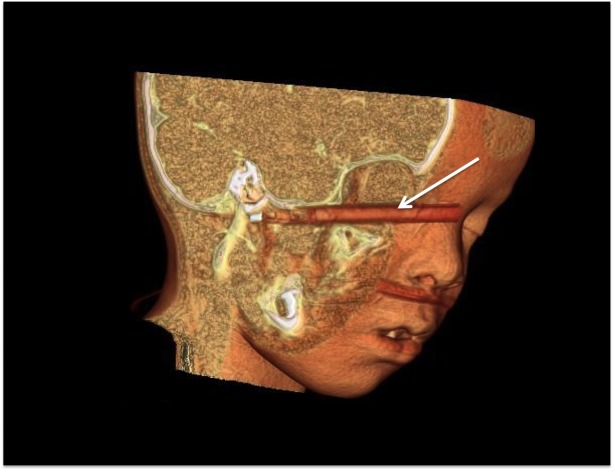
3D CT reconstruction of a child who fell on a writing pen. The pen entered the right orbit, then followed the skull base to end just anterior to the internal carotid artery. 3D CT: three-dimensional computed tomography

Penetrating injuries to the orbit are rare but have the potential to cause mortality and severe morbidity (Figures 1-3). Surprisingly, in many cases of penetrating injuries to the orbit, clinical signs are not immediately apparent [1]. Because of the close proximity of the orbit to many distinct structures, any penetration injury requires a multidisciplinary follow-up. In many cases, ophthalmologists, neurosurgeons, otolaryngologists, maxillofacial surgeons, and radiologists need to be involved for appropriate patient care.

Penetrating orbital injuries (POIs) can be either missile or non-missile depending on the speed of the object. Missile penetrating objects travel at > 100 meters/second and tissue injury is mediated mainly by heat. In non-missile penetrating injuries, the penetrating object moves < 100 meters/second, and tissue injury is dictated by the lacerative potential of the object. This review focuses on non-missile POI. In addition to pens and pencils, an unusual array of penetrating objects have been reported in the literature, including umbrellas, knives, chopsticks, eyeglasses, fern, toilet brush handles, forks, keys, knitting needles, and shelving bars. Falls, suicide attempts, and assaults have all been reported causes of these POIs [2]. 

## Review

Presentation of POIs depends on the nature, orientation, and depth of the penetrating object. Because the orbit is a quadrilateral pyramid, an object can penetrate the medial, lateral, superior, or inferior rim. Less commonly, extraorbital penetration can occur, in which an object enters the orbit from the posterior aspect (i.e., penetrates the neck and enters the orbit).

Anatomy

The globe occupies approximately one-fifth of the orbit. The rest of the orbit comprises muscles, vessels, and nerves within a matrix of orbital fat. The superior rim of the orbit is formed by the frontal bone. As such, the frontal sinus resides on the medial aspect of the roof, and the fossae for the lacrimal glands reside on the lateral aspect of the roof. The roof of the orbit also serves as the floor of the anterior cranial fossa. The medial wall of the orbit consists mainly of the ethmoid bone, but components of the palatine and lacrimal bones are also present. The maxilla, which is the most commonly fractured orbital bone, forms the floor of the orbit. Lastly, the lesser wing of the sphenoid forms the lateral wall of the orbit. There are two orbital fissures and one canal at the apex of the orbit. The optic canal is formed by the sphenoid bone and allows communication from the orbit to the middle cranial fossa. The optic nerve and ophthalmic artery normally traverse the canal, but there is a potential for foreign objects to penetrate the canal and enter the middle cranial fossa. The fissure between the greater and lesser wings of the sphenoid forms the superior orbital fissure. The oculomotor, trochlear, ophthalmic, and abducens nerves all traverse this fissure. A foreign object lodged in this region will injure these nerves and can enter the cranial cavity, resulting in orbital apex syndrome. The maxilla and the greater wing of the sphenoid form the inferior orbital fissure. This fissure allows communication between the orbit and the pterygopalatine fossa and infratemporal fossa. The superior orbital fissure and optic canal can allow a penetrating object to enter the cranial cavity without breaking any of the orbital bones [3].

Entry modes

As evident by the anatomy surrounding the orbit, the presentation of POI will depend on the orientation and point of entry of the foreign object. Although there is enormous literature regarding POI, no author has organized POI and its presentation on the basis of entry mode. This organization will allow the clinician to approach POIs more systematically.

Medial Rim of the Orbit

Assault, suicide attempts, and accidental penetration account for a vast number of medial POIs [1-2, 4-5]. Objects that enter the medial rim of the orbit mostly enter the cranial cavity through the superior orbital fissure [4-6], but there is potential to enter the optic canal [5]. These objects pass through the medial aspect of the superior orbital fissure and enter the medial aspects of the middle cranial fossa just lateral to the sella turcica and the cavernous sinus [2]. Depending on the depth of penetration, the trajectory can continue just lateral to the pons and the basilar cisterns [2, 4-5]. This trajectory through the orbit injures the medial and inferior rectus muscles [5]. Patients also commonly develop orbital apex syndrome, which is defined as the absence of light perception due to optic nerve injury, ipsilateral superior orbital fissure nerve palsies resulting in ophthalmoplegia, oculosympathetic nerve paralysis, and lastly, sensory loss of the ophthalmic division of the trigeminal nerve [4].

As Schreckinger and colleagues discuss, objects entering the superior orbital fissure have the potential to course through the cavernous sinus and penetrate the brainstem, which can be life-threatening. Patients can also present with cavernous sinus syndrome, which presents similarly to orbital apex syndrome with the addition of facial numbness and miosis. Entry into the cranial cavity via the optic canal will position the penetrating object near the internal carotid artery and the optic nerve at the level of the suprasellar cistern [2].

Less commonly, objects can enter through the medial rim of the orbit and course superiorly as opposed to posteriorly. In such cases, the frontal bone is fractured and the object gains entry into the anterior cranial fossa and can traverse as far as the parietal lobe [1, 7].

Superior Rim of the Orbit

Falls account for a vast number of orbital roof POIs [8-9]. Penetration through the superior rim of the orbit can be notoriously difficult to detect, and sometimes only lid laceration serves as the only clue to injury [9]. Penetration of the orbital roof most often results in frontal lobe contusion [2, 8-9] because the frontal bone is very thin [2]. In one case [9], leakage of clear fluid from the superior eyelid was the only exterior manifestation of the POI in a toddler. Analysis for beta-tracer-protein indicated that the fluid was cerebrospinal fluid and prompted an imaging workup.

Inferior Rim of the Orbit

Cases in which foreign objects penetrate the floor of the orbit are relatively rare and usually involve assault. One stabbing victim had a knife lodged in the inferior aspect of his orbit, lacerating the inferior eyelid, traversing the maxillary sinus, penetrating the soft palate, and lacerating the left tonsil. Removal of the knife was uneventful and without complications. The patient reported numbness over the maxilla attributed to laceration of the infraorbital nerve and diplopia with upper-lateral gaze attributed to reactive edema [10].

Lateral Rim of the Orbit

Cases with objects penetrating the lateral rim of the orbit are not reported in the literature. As Lasky and colleagues suggest, avoidance head turns will generally direct penetrating objects to the medial aspect of the orbit, especially in self-inflicting cases [4]. Furthermore, we hypothesize that the 45° difference between the lateral orbital wall and the sagittal plane would make it difficult for penetrating objects to be directed deep into the orbit.

Extraorbital Entry

Extraorbital entry is not a common POI and only a few cases have been reported in the literature. These types of injuries can vary drastically in presentation depending on the point of entry and the course of the object before it enters the orbit. One case involved a drowsy driver of a motor vehicle being impaled by the windshield wiper control indicator. The control indicator traversed the right maxilla, maxillary sinus, the nasal cavity and turbinates, the ethmoid sinuses bilaterally, and the superomedial aspect of the left orbit. The patient suffered temporary ophthalmoplegia and permanent blindness in the left eye [2].

Complications

Complications associated with specific modes of entry have already been discussed. Complications that are common to all POIs are discussed here.

Sympathetic ophthalmia is a bilateral necrotizing granulomatous uveitis that results from trauma to the globe and can accompany any type of POI. POI and postoperative complications include CSF leaks, traumatic aneurysm, cavernous fistula, cerebral abscess, and meningitis [2]. The risk for abscess formation increases exponentially when the penetrating object is organic. For example, the organic and highly porous nature of a wooden pencil allows bacteria to thrive and can act as an infective nidus if small pieces remain intracranially [6, 11].

Imaging and diagnosis

Noncontrast CT scanning is the preferred imaging modality for determining the course of the penetrating object and the extent of tissue injury. MRI is useful when the penetrating object is wooden because the foreign object can easily be differentiated from the surrounding tissue. With CT scanning, dry wood has a similar density to air and wet wood has a similar density to adjacent tissue. If there is hemorrhage or injury to a blood vessel is suspected, angiography is indicated [2].

Treatment

A full radiological workup must precede removal of the foreign object. Although rare, there have been cases of fatal hemorrhage in uncontrolled environments. Schreckinger and colleagues suggest initiation of antibiotic therapy upon admission of the patient. Patients who have not suffered orbital bone fractures can generally be treated conservatively. Surgical intervention becomes necessary when the foreign object is retained in the orbit or there are bone fractures, CSF leaks, intracranial hematomas, or vascular injuries. Any surgical plans require a multidisciplinary effort involving ophthalmologists, neurosurgeons, otolaryngologists, maxillofacial surgeons, and radiologists for proper care [2].

## Conclusions

Although POIs represent only a small portion of head injuries, they require multidisciplinary care. They can be classified on the basis of the orbital rim of entry. Each rim is associated with a characteristic set of complications. By determining the mode of entry and understanding the complications associated with each mode, a clinician can predict the clinical course of a patient with a POI more accurately.
